# *Leptochilus yangjiangensis* (Polypodiaceae), a New Fern Species From Guangdong, China

**DOI:** 10.1002/ece3.73621

**Published:** 2026-05-14

**Authors:** Hua‐Jing Zhou, Ting Wang, Yi Huang, Yu‐Han Fang, Bin Zhang, Guo‐Di Chen, Hong‐Feng Chen, Fa‐Guo Wang

**Affiliations:** ^1^ Guangdong Provincial Key Laboratory of Applied Botany, State Key Laboratory of Plant Diversity and Specialty Crops, South China Botanical Garden Chinese Academy of Sciences Guangzhou China; ^2^ University of Chinese Academy of Sciences Beijing China; ^3^ Yunnan Key Laboratory of Plateau Wetland Conservation, Restoration and Ecological Services Southwest Forestry University Kunming China; ^4^ Yangjiang Municipal People’s Government Taiwan Hong Kong and Macao Affairs Bureau Yangjiang China

**Keywords:** *Leptochilus*, morphology, new species, phylogenetic analysis, plastid genome

## Abstract

A new fern species, *Leptochilus yangjiangensis* (Polypodiaceae), was discovered during field surveys in Ziluo Mountain, Yangjiang City, Guangdong Province, China. This study provides a detailed morphological description and illustrations of the new species. Morphologically, this species resembles *Leptochilus pedunculatus* and *L. ovatus* in plant height, petiole morphology, and soral morphology, but differs from them in its coriaceous laminae and lanceolate fertile fronds. To confirm its taxonomic status, phylogenetic analyses based on three plastid genome regions (*rbcL, trnL‐F, and rps4+rps4‐trnS*) indicated that the new species forms a distinct and well‐supported monophyletic clade and is sister to *L. dolichophyllus*. Furthermore, the complete plastid genome of this new species is reported for the first time. Preliminarily assessed as Data Deficient (DD) according to IUCN guidelines, this new species enriches the diversity of *Leptochilus*.

## Graphical abstract

We describe *Leptochilus yangjiangensis*, a new fern species from Guangdong, China, which was previously misidentified as *L. pedunculatus* ; it differs from morphologically similar congeners by coriaceous laminae and lanceolate fertile fronds. Phylogenetic analyses (using three plastid regions) place it as sister to L. dolichophyllus (forming a well‐supported clade), and we report its complete plastid genome for the first time. This discovery corrects a long‐standing taxonomic error, expands Leptochilus diversity, and provides preliminary insights into the genus's species delimitation and distribution patterns in southern China.

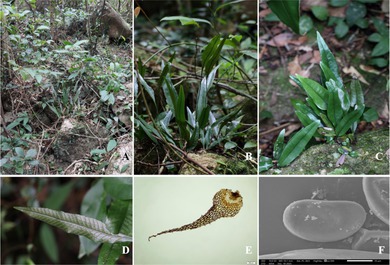

## 1 Introduction

*Leptochilus* Kaulfuss (1824: 147) is a group of small to medium‐sized ferns belonging to the family Polypodiaceae. It is widely distributed in tropical regions of Asia, with a few species extending to Pacific islands. In China, the genus is mainly concentrated in South China, Southwest China, and Taiwan, serving as an important component of the Asian fern flora. Owing to pronounced morphological variation among species, the taxonomic delimitation of *Leptochilus* has long been problematic, leading to persistent confusion and repeated revisions of species circumscription.

Originally described as a small genus containing only a single species, *Leptochilus* was later estimated to include approximately 25 species (Zhang et al. 2015). However, species boundaries remained ambiguous, and numerous closely related taxa were independently classified rather than being assigned to this genus. Currently, based on integrative taxonomic studies including phylogenetic analyses of six plastid markers and the nuclear *pgiC* gene, with a sampling size of 226 accessions covering more than 70 taxa, Zhang, Liang, et al. (2024) and Zhang, Lu, et al. (2024) have confirmed that *Leptochilus* comprises 51 species.

Morphologically, *Leptochilus* is characterized by considerable diversity, with species exhibiting terrestrial, epiphytic, or lithophytic growth habits, reflecting broad ecological adaptability. The leaves are monomorphic or dimorphic, with palmate, digitate, pinnatifid, or pinnate compound forms; sori are orbicular, elongate to linear.

During recent field surveys conducted in Ziluo Mountain, Yangjiang City, Guangdong Province, China, a unique fern caught our attention. Preliminary morphological observations indicated that it could not be assigned to any known species of *Leptochilus*. Through comprehensive morphological and phylogenetic studies, we confirm that this species represents an undescribed new taxon and describe it here as *L. yangjiangensis*.

## 2 Materials and Methods

### 2.1 Morphological Study

This study first adopted the *Leptochilus* species identification key constructed by Zhang, Liang, et al. (2024) and Zhang, Lu, et al. (2024) for preliminary taxonomic screening, and then retrieved and collated specimen information of related taxa from digital databases, including the Chinese Virtual Herbarium (CVH, https://www.cvh.ac.cn/), the Global Biodiversity Information Facility (GBIF, https://www.gbif.org/), and Global Plants on JSTOR (https://plants.jstor.org/). Based on these, field‐collected specimens of *L. yangjiangensis* were used for comparative morphological analyses with its similar species ( *L. pedunculatus* : isotypes E00507825, E00507826; *L. ovatus* : holotype MICH 1190696, isotype BM 000036785) and the closely related species *L. dolichophyllus* (Fu et al. 2025). The overall morphology and habitat of *L. yangjiangensis* were photographed using a digital camera (Canon EOS R). Morphological data of sterile and fertile fronds of *L. yangjiangensis* (specimen no. IBSC 1094808, deposited at the South China Botanical Garden Herbarium, Guangzhou, China) were measured using MATO (Liu et al. 2023). Scale color and other key traits were observed using a stereomicroscope (OLYMPUS‐SZ61) and a biological microscope (OLYMPUS‐BX43), and spore ornamentation characteristics were obtained using a Scanning Electron Microscope (JSM‐IT210LV).

### 2.2 Taxon Sampling and Sequencing

Leaves from two different individuals of the new species were collected and dried in silica gel. Total DNA was extracted using the cetyltrimethylammonium bromide (CTAB) method (Allen et al. 2006). Genomic DNA was fragmented to approximately 350 bp, followed by end repair, 3′ adenylation, adapter ligation, fragment selection, and PCR amplification to construct a sequencing library. After quality control, the library was sequenced on the Illumina Novaseq platform with paired‐end 150 bp (PE150) reads, and raw data were filtered for low‐quality sequences using fastp before subsequent analyses (Yan et al. 2013).

### 2.3 Plastome Assembly and Annotation

The cleaned reads were de novo assembled into plastome contigs using the GetOrganelle pipeline (v1.7.5+, https://github.com/Kinggerm/GetOrganelle; Jin et al. 2020). To ensure accuracy, the assembled plastome was visually inspected and edited with Bandage v.0.8.1 (Wick et al. 2015) to resolve ambiguities, yielding a complete circular genome. Annotation was performed in Geneious v.11.1.5 (Kearse et al. 2012) with reference to the plastome sequence of the closely related species *Leptochilus hemionitideus* (NC_040177.1).

### 2.4 Phylogenetic Analyses

Maximum Likelihood (ML) and Bayesian Inference (BI) methods were used for molecular phylogenetic analyses. According to Zhang et al. (2020), target genes were extracted from chloroplast genomes, including 43 *rbcL*, 42 *trnL‐F*, and 44 *rps4+rps4‐*trnS, which were downloaded from GenBank for 46 taxa to construct phylogenetic trees (Table 1). Sequence alignment was performed using MAFFT v.7.475 (Katoh and Standley 2013), and regions with low alignment quality were trimmed using trimAl (Capella‐Gutiérrez et al. 2009) to optimize the dataset. Phylogenetic trees were constructed using two methods: Maximum Likelihood (ML) and Bayesian Inference (BI). The optimal evolutionary models for ML and BI were selected using ModelTest‐NG (Darriba et al. 2020) under the Bayesian Information Criterion (BIC), with the parameter “‐T raxm1” specified for ML and “‐T mrbayes” for BI.

**TABLE 1:** Voucher and GenBank accession information of species used for plastid genome assembly. A dash (—) indicates missing data.

Scientific name	Voucher	*rbcL*	*trnL‐F*	*rps4 + rps4‐trnS*
*Leptochilus bolikhamsaiensis* Liang Zhang, Thepkaysone & Z. Zhou	Zhuo Z. et al. LZ156 (KUN)	PQ316915	PQ317022	PQ316962
*Leptochilus cantoniensis* (Baker) Ching	Dong S.‐Y. 1034 (IBSC)	EU363245	—	EU363258
*Leptochilus* cf. *ellipticus*	Chen C.‐C. 1065 (H)	MH665038	MH665169	MH665102
*Leptochilus* cf. *flexilobus*	Zhang L.‐B. et al. 6710 (CDBI, MO, VNMN)	MH768417	MH768545	MH768479
*Leptochilus* cf. *hemitomus*	Zhang L.‐B. et al. 6484 (CDBI, MO, VNMN)	MH768427	MH768555	MH768489
*Leptochilus* cf. *pothifolius*	Cicuzza D. 1998 (HITBC)	PQ316873	PQ317004	PQ316943
*Leptochilus chingii* Liang Zhang & Li Bing Zhang	Zhang L.‐B. et al. 7453 (CDBI, MO, VNMN)	MH768437	MH768565	MH768502
*Leptochilus daklakensis* Liang Zhang, T. T. Luong, X. M. Zhou & Li Bing Zhang	Zhang L.‐B. et al. 8944 (CCDBI)	PQ316871	PQ317002	PQ316941
*Leptochilus digitatus* (Baker) Noot.	A.R. Smith 00–036 (UC)	EU482948	EU483044	EU482998
*Leptochilus dissimilialatus* (Bonap.) Liang Zhang & Li Bing Zhang	Zhang L.‐B. et al. 6362 (CDBI, MO, VNMN)	MH768419	MH768547	MH768481
*Leptochilus dolichophyllus* H. H. Fu & H. J. Wei	She‐Lang Jin, Hou‐Hua Fu JSL9400	PV442128	PV442128	PV442128
*Leptochilus flexilobus* (Christ) Liang Zhang & Li Bing Zhang	Zhang L. et al. 3050 (KUN)	PQ316887	PQ317020	PQ316960
*Leptochilus gracilis* Z. L. Liang, Liang Zhang & Li Bing Zhang	Liang Z.‐L. et al. 607 (KUN, CDBI)	—	MW142228	MW142229
*Leptochilus hemionitideus* (C. Presl) Noot.	Wu S.‐K. et al. WS‐2437 (KUN)	JX103694	JX103778	JX103736
*Leptochilus hemitomus* (Hance) Noot.	Zhang X.‐C. 3302 (PE)	EU482951	EU483047	EU483001
*Leptochilus henryi* (Baker) X. C. Zhang	DJY04047 (CDBI)	MH768428	MH768556	MH768490
*Leptochilus heterophyllus* (S. K. Wu & K. L. Phan) Christenh.	Wu S.‐K. et al. WP‐136 (KUN)	JX520933	JX520937	JX520935
*Leptochilus kachinensis* Liang Zhang & Li Bing Zhang	Deng Y.‐F. et al.3200 (CDBI)	PQ316865	PQ316996	PQ316936
*Leptochilus khammouanensis* Liang Zhang, Thepkaysone & Z. Zhou	Shui Y.M. et al. LK136 (KUN)	PQ316872	PQ317003	PQ316942
*Leptochilus leveillei* (Christ) X. C. Zhang & Noot.	Zhang L.‐B. et al. 475 (CDBI)	PQ316867	PQ316998	PQ316938
*Leptochilus leveillei* (Christ) X. C. Zhang & Noot.	Zhang X.‐C. 4312 (PE)	MH665047	MH665179	MH665112
*Leptochilus leveillei* (Christ) X. C. Zhang & Noot.	Zhang L.‐B. et al. 6623 (CDBI)	—	PQ316990	PQ316929
*Leptochilus leveillei* (Christ) X. C. Zhang & Noot.	Zhang L.‐B. et al. 7069 (CDBI)	PQ316861	PQ316991	PQ316930
*Leptochilus luangprabangensis* Liang Zhang, Thepkaysone & Z. Zhou	Zhuo Z. et al. LZ415 (KUN)	PQ316898	PQ317034	PQ316974
*Leptochilus macrophyllus* (Blume) Noot.	Wade 1772 (TAIF)	MH768447	MH768573	MH768511
*Leptochilus morsei* (Ching) Fraser‐Jenk.	Jin S.‐L. et al. JSL7322 (CSH)	—	PQ317014	PQ316954
*Leptochilus multilobus* Liang Zhang & X. M. Zhou	YLZB2004 (CDBI)	PQ316868	PQ316999	—
*Leptochilus oblongus* Li Bing Zhang, Liang Zhang & N. T. Lu	Zhang L.‐B. et al. 6299 (CDBI, MO, VNMN)	MH768429	MH768557	MH768491
*Leptochilus ovatifolius* Zhe Zhang, S. W. Yao & Yi Huang	Zhang L. NAS20180929_4 (KUN)	PQ316880	—	PQ316951
*Leptochilus ovatus* Copel.	Wade 1583 (TAIF)	PQ316863	PQ316994	PQ316934
*Leptochilus pedunculatus* (Hook. & Grev.) Fraser‐Jenk.	Zhang L. et al. 2758 (KUN)	PQ316917	PQ317024	PQ316964
*Leptochilus pedunculatus* (Hook. & Grev.) Fraser‐Jenk.	Zhang L. et al. 4470 (KUN)	PQ316907	PQ317043	PQ316981
*Leptochilus pedunculatus* (Hook. & Grev.) Fraser‐Jenk.	Zhang L. et al. 2855 (KUN)	PQ316882	PQ317012	PQ316952
*Leptochilus pentaphyllus* (Baker) Liang Zhang & Li Bing Zhang	Xu C.‐D. A0357 (PE)	MH665043	MH665175	MH665108
*Leptochilus pteropus* (Blume) Fraser‐Jenk.	Zhang L.‐B. et al. 8014 (CDBI, MO, VNMN)	MH768412	MH768540	—
*Leptochilus sanjiangensis* (H. G. Zhou & Hua Li) Liang Zhang & Li Bing Zhang	Jin S.‐L. et al. JSL7568 (CSH)	PQ316889	PQ317025	PQ316965
*Leptochilus saxicola* (H. G. Zhou & H. Li) Liang Zhang & Li Bing Zhang	Zhang L.‐B. et al. 6772 (CDBI, MO, VNMN)	MH768441	MH768569	MH768505
*Leptochilus scandens* H. J. Wei & Yi Huang	Jin S.‐L. et al. JSL8000 (CSH)	PQ316911	—	PQ316985
*Leptochilus × shintenensis* (Hayata) X. C. Zhang & Noot.	Knapp R. 3874 (P)	MH768454	—	MH768518
*Leptochilus* sp.	Knapp R. 3849 (P)	MH768443	MH768571	MH768507
*Leptochilus wrightii* (Hook. & Baker) X. C. Zhang	Li 47 (PE)	MH665064	MH665197	MH665130
*Leptochilus wusugongii* Liang Zhang & Li Bing Zhang	Wu S.‐K. et al. WS‐2591 (KUN)	PQ316874	PQ317005	PQ316944
***Leptochilus yangjiangensis* F. G. Wang, Y. Huang & H. J. Zhou**	Yi Huang, Guo‐Di Chen HY2868 (IBSC)			
Outgroup				
*Microsorum commutatum* (Blume) Copel.	Wade 3768 (TAIF)	MH051171	MH113503	MH113470
*Microsorum insigne* (Blume) Copel.	Liu 204 (PE)	EU482957	EU483054	EU483008
*Microsorum punctatum* (L.) Copel.	Wade 1390 (TAIF)	MH051177	MH113509	MH113476
*Phymatosorus longissimus* (Blume) Pic. Serm.	Cheng X. et al. FB042 (KUN)	MT130640	MT130640	MT130640

*Note:* Bold text indicates the new species described in this study.

ML analysis was implemented in RAxML v.8.2.10 (Stamatakis 2014), and node support values were evaluated via the Rapid Bootstrap Support (RBS) method with 1000 pseudoreplicates. BI analysis was conducted using MrBayes v.3.2 (Ronquist et al. 2012) with 1,000,000 generations, and trees were sampled every 100 generations.

## 3 Results

### 3.1 Characteristics of the Plastid Genome

**FIGURE 1:**
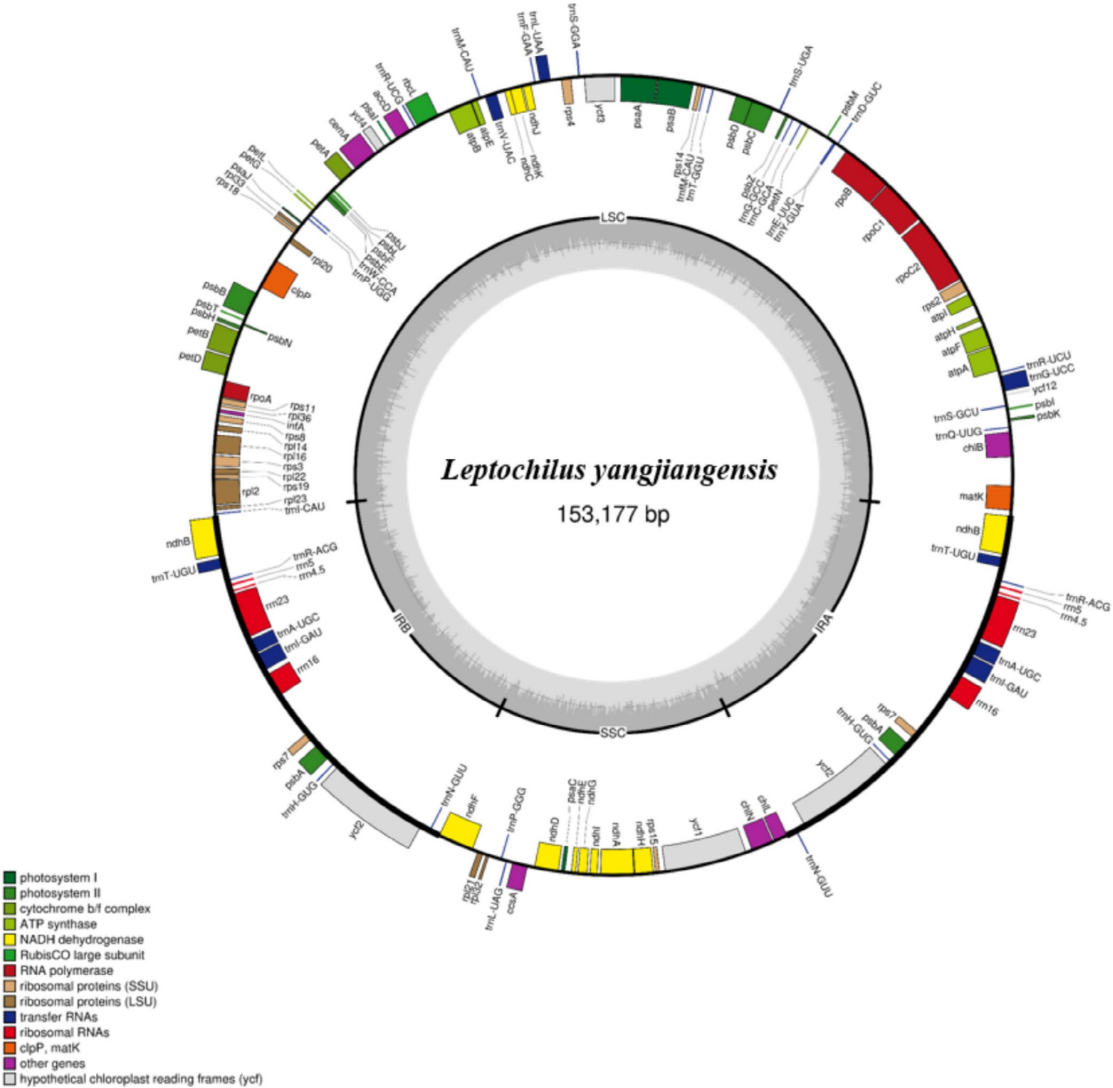
Plastome annotation map of *Leptochilus yangjiangensis*. The darker gray in the inner circle corresponds to GC content. Outside the GC content region, IRA and IRB (two inverted repeat regions); LSC (large single‐copy region); and SSC (small single‐copy region) are indicated.

We successfully assembled the complete plastid genome of *Leptochilus yangjiangensis*, with a total length of 153,177 bp and a GC content of 43.9% (Figure 1). The plastid genome exhibits a typical quadripartite structure, consisting of a pair of inverted repeat (IR) regions (24,880 bp), a large single‐copy (LSC) region (81,525 bp), and a small single‐copy (SSC) region (21,892 bp). The plastid genome of *L. yangjiangensis* encodes 115 genes, including 82 protein‐coding genes, 29 transfer RNA (tRNA) genes, and four ribosomal RNA (rRNA) genes.

### 3.2 Phylogenetic Relationships

**FIGURE 2:**
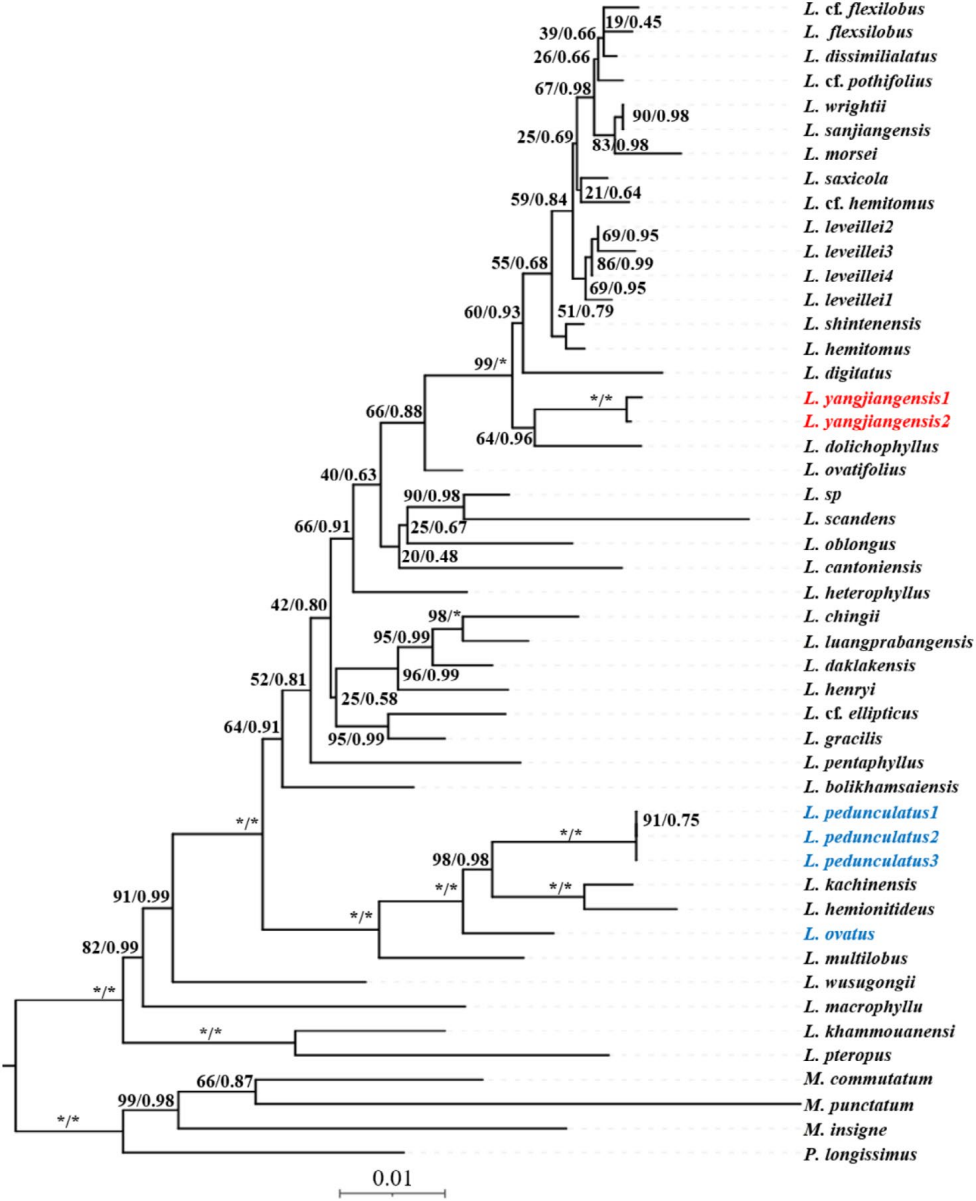
Maximum Likelihood (ML) and Bayesian Inference (BI) phylogenetic tree constructed based on plastid genome regions (*rbcL*, *trnL‐F*, and *rps4+rps4‐trnS*). Numbers adjacent to nodes represent ML bootstrap support values (BS) and BI posterior probabilities (PP) (presented as “BS/PP”). The asterisk (*) at nodes indicates 100% bootstrap support (for ML) and 1.00 posterior probability (for BI). Red text denotes the new species, whereas blue text denotes the species morphologically similar to the new species.

Using *Microsorum commutatum*, *M. insigne* , *M. punctatum* , and *Phymatosorus longissimus* as the outgroup, phylogenetic analyses based on three molecular markers showed that the newly discovered *Leptochilus yangjiangensis* forms a distinct, strongly supported clade, and is sister to *L. dolichophyllus* (Figure 2). In addition, although *L. yangjiangensis* is morphologically similar to *L. pedunculatus* and *L. ovatus* , the phylogenetic results indicate that it is only distantly related to these two species.

## 4 Taxonomic Treatment

**FIGURE 3:**
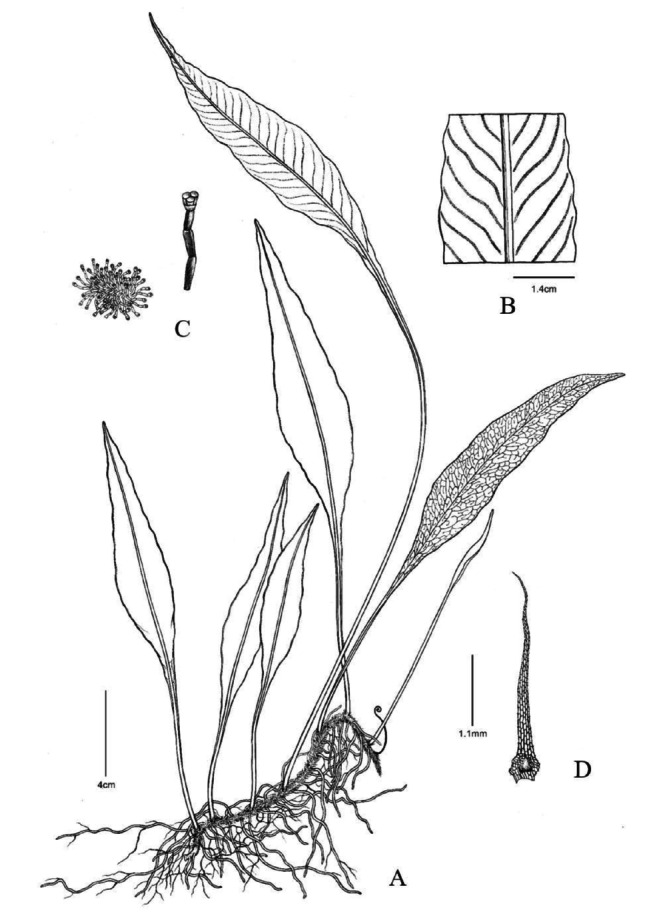
Line drawings of *Leptochilus yangjiangensis*. (A) Habit. (B) Portion of fertile frond showing sori. (C) Sori and paraphyses. (D) Scale. These illustrations were drawn by Yun‐Xiao Liu.

**FIGURE 4:**
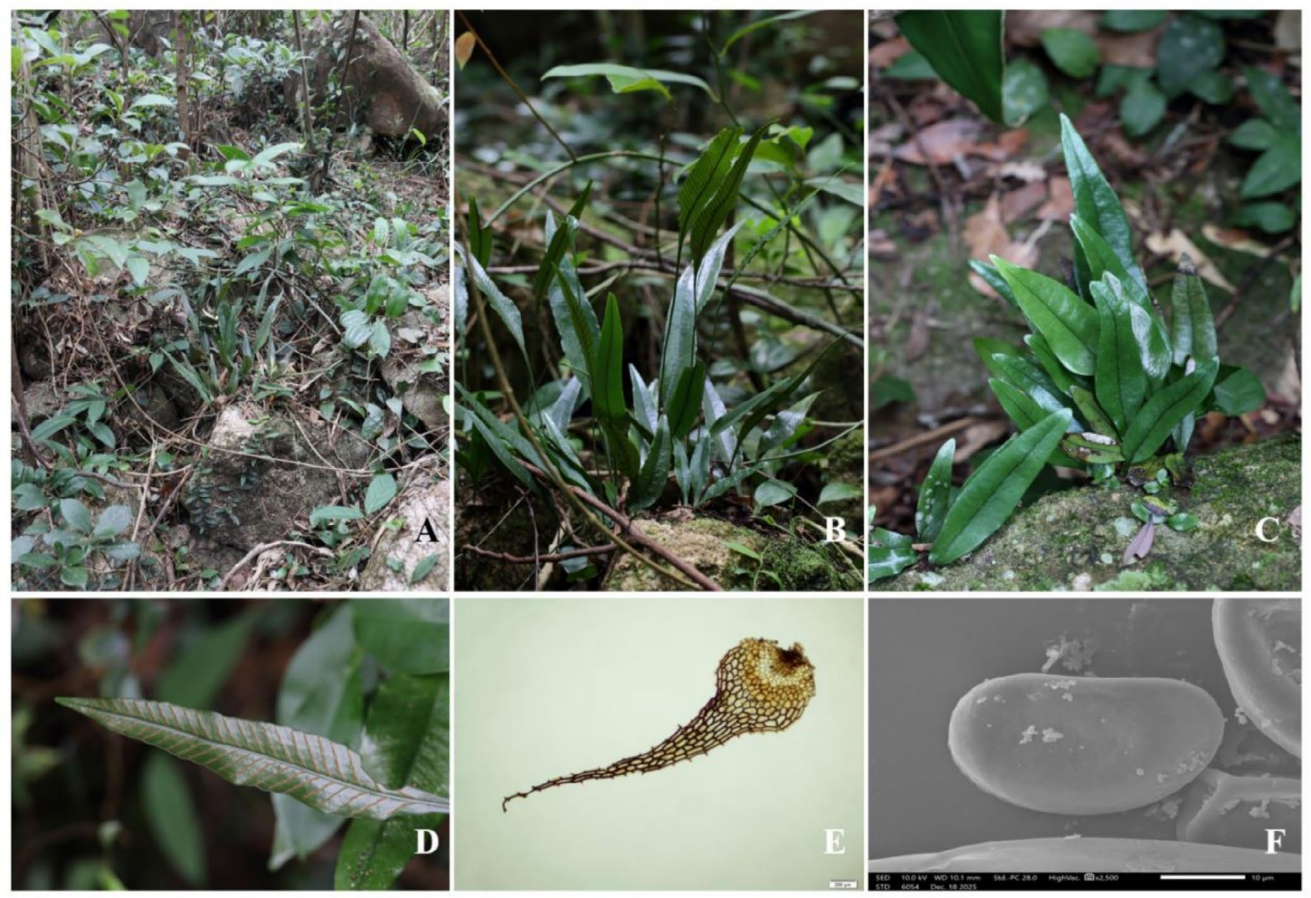
Habitat and morphology of *Leptochilus yangjiangensis*. (A) Habitat. (B) Whole plant. (C) Habit. (D) Close‐up of fronds with sori. (E) Scale structure under a biological microscope. (F) Spore morphology under a scanning electron microscope. Photographs by Guo‐Di Chen (A–D).

*Leptochilus yangjiangensis* F. G. Wang, Y. Huang & H. J. Zhou, sp. nov. (Figures 3 and 4).

*Type:* CHINA. Guangdong Province: Ziluo Mountain, Xinzhou Town, Yangdong District, Yangjiang City, 21°49′34″ N, 112°22′41″ E, ca. 600 m elev., 10 October 2025, Yi Huang, Guo‐Di Chen HY2868 (Holotype: IBSC!).

### 4.1 Diagnosis

*Leptochilus yangjiangensis* is similar to both *L. pedunculatus* and *L. ovatus* , but can be distinguished by the following diagnostic characters (Table 2): (1) lamina coriaceous (vs. herbaceous in both *L. pedunculatus* and *L. ovatus* ); (2) fertile fronds lanceolate (vs. ovate‐lanceolate in both *L. pedunculatus* and *L. ovatus* ); (3) life form lithophytic (vs. lithophytic in *L. pedunculatus* and hemiepiphytic in *L. ovatus* ). Additionally, it can be distinguished from its sister species *L. dolichophyllus* by the smaller plant size (fertile fronds 26.0–43.0 cm long vs. 43.0–60.0 cm long in *L. dolichophyllus*) and distinct life form (lithophytic vs. hemiepiphytic in *L. dolichophyllus*).

**TABLE 2:** Morphological comparison with similar and closely related species.

Characters	*Leptochilus yangjiangensis*	*Leptochilus pedunculatus*	*Leptochilus ovatus*	*Leptochilus dolichophyllus*
Life form	Lithophytic	Lithophytic	Hemiepiphytic	Hemiepiphytic
Frond dimorphism	Dimorphic	Subdimorphic	Subdimorphic	Subdimorphic
Lamina texture	Coriaceous	Herbaceous	Herbaceous	Herbaceous
Fertile frond shape	Lanceolate	Ovate‐lanceolate	Ovate‐lanceolate	Linear to linear lanceolate

### 4.2 Description

Plants perennial, evergreen, lithophytic. Rhizome creeping, densely rooted, uniformly covered with scales throughout; scales brown, ovate‐lanceolate, 2.3–3.1 × 0.8–1.0 mm, apex acuminate, margins sparsely serrate. Fronds distant, distinctly dimorphic, coriaceous, glabrous on both surfaces; sterile fronds: 8.0–20.0 cm long; stipe 3.0–79.0 mm long; lamina lanceolate, 8.0–26.0 × 0.7–2.5 cm, apex acuminate to shortly acuminate. Fertile fronds: 14.0–22.0 cm long, reaching 9.0–25.0 cm; lamina lanceolate, 26.0–43.0 × 1.2–2.1 cm, apex long acuminate; adaxially dark green, abaxially pale green. Fertile fronds are approximately twice as long as sterile fronds. Sori linear, borne on anastomosing veins, 1 regular row between each pair of lateral veins, obliquely emerging from midrib, most extending to leaf margin. Spores reniform, 21.7–36.7 × 13.5–21.3 μm.

### 4.3 Additional Specimens Examined

CHINA. Guangdong: Xinhui, Gudoushan Nature Reserve, ca. 450 m elev., 3 January 2002, Yue‐Hong Yan (HUST 00001462). Guangdong: Jiangmen City, Taishan City, Beidou Town, Mount Ziluo, ca. 420 m elev., 14 December 2025, Guo‐Di Chen (IBSC 1095498). Guangdong: Jiangmen City, Taishan City, Beidou Town, Mount Ziluo, ca. 420 m elev., 14 December 2025, Guo‐Di Chen (IBSC 1095499). Guangdong: Jiangmen City, Taishan City, Beidou Town, Mount Ziluo, ca. 420 m elev., 14 December 2025, Guo‐Di Chen (IBSC 1095500). Guangdong: Jiangmen City, Taishan City, Beidou Town, Mount Ziluo, ca. 420 m elev., 14 December 2025, Guo‐Di Chen (IBSC 1095501).

### 4.4 Distribution and Habitat

*Leptochilus yangjiangensis* occurs on mixed forest slopes along ravines in the coastal regions from Yangjiang City to Taishan City, Guangdong Province, China.

### 4.5 Etymology

The specific epithet yangjiangensis refers to Yangjiang City, Guangdong Province, China, the type locality of this species.

### 4.6 Vernacular Name

阳江线蕨 (yang jiang xian jue).

### 4.7 Conservation Status

Approximately 200 mature individuals of *Leptochilus yangjiangensis* have been observed in Mount Ziluo, Yangjiang City, Guangdong Province, China. This species has also been found in the coastal mountain forests of Taishan City, Guangdong Province, yet the population size at this locality remains unconfirmed. In view of the existing gaps in population data and insufficient distribution information for this species, it is recommended to be categorized as Data Deficient (DD) following the IUCN Red List Categories and Criteria (IUCN 2024).

### 4.8 Key to Morphologically Similar or Phylogenetically Related Species to *Leptochilus yangjiangensis*

1 Terrestrial or lithophytic…………2

+ Hemiepiphytic…………3

2(1) Laminae coriaceous; fertile laminae lanceolate…………*L. yangjiangensis*

+ Laminae herbaceous; fertile laminae oblong to broadly oblong…………*L. pedunculatus*

3(1) Fertile laminae linear to linear‐lanceolate…………*L. dolichophyllus*

+ Fertile laminae ovate to narrowly ovate………… *L. ovatus*

## 5 Discussion

As an important group of Polypodiaceae, *Leptochilus* has long suffered from ambiguous taxonomic delimitation and specimen identification errors due to strong morphological plasticity and overlapping diagnostic characters among some species (Dong et al. 2008; Zhang et al. 2019). The new species described herein has long been misidentified as *Leptochilus pedunculatus* in southern China. However, recent studies by Zhang, Liang, et al. (2024) and Zhang, Lu, et al. (2024) have revealed that the natural distribution of *L. pedunculatus* may be restricted to low‐altitude areas of the Himalayas, while the discovery site of this new species is the coastal mountains of Yangjiang City, Guangdong Province, China. The currently known geographical distributions of the two species show no overlap, suggesting that they are unlikely to be conspecific from a biogeographic perspective. Integrating evidence from molecular phylogenetics, plastid genome data, and morphology, this study confirms that the species is an independent new species of *Leptochilus*, which not only corrects long‐standing taxonomic misplacement but also provides key supplements for understanding the species diversity and distribution pattern of the genus in southern China.

Molecular evidence has become an important basis for modern species delimitation and confirmation of the evolutionary independence of new species. Phylogenetic analyses based on molecular markers show that the new species forms a highly supported monophyletic clade (BS = 100), and its evolutionary branch is clearly separated from other congeneric species, confirming its independent evolutionary status. The phylogenetic tree also reveals that *Leptochilus yangjiangensis* forms a sister group relationship with *Leptochilus dolichophyllus*. The complete plastid genome of *L. yangjiangensis* successfully obtained in this study further strengthens the molecular evidence chain. Its genomic structure is consistent with the conserved characteristics of previously published *Leptochilus* plastid genomes (Fu et al. 2025; Min et al. 2018), verifying its correct affiliation at the generic level. This is consistent with the conclusion of Zhang et al. (2019) that plastid genome sequences can effectively resolve the relationships among species within *Leptochilus*, highlighting the important value of genomic data in addressing the taxonomic ambiguity of this genus.

Although *L. yangjiangensis* is closely related to *L. dolichophyllus* phylogenetically, nevertheless it exhibits significant morphological differentiation and clear biogeographic isolation. The taxonomic clarification of *L. yangjiangensis* is of great significance for the revision of *Leptochilus*. The long‐term misidentification of *L. yangjiangensis* as *L. pedunculatus* indicates that morphological similarity within *Leptochilus* is prone to classification errors, and it is urgent to reevaluate herbarium collections and regional floristic records. Meanwhile, the discovery of this new species expands the known diversity of *Leptochilus* in southern China, suggesting that there may still be undiscovered cryptic species in subtropical and tropical Asia.

In conclusion, based on integrative evidence from phylogenetics, plastid genomics, morphology, and biogeography, this study confirms that *Leptochilus yangjiangensis* is an independent taxon. This research not only corrects long‐standing taxonomic misplacement but also provides new perspectives for understanding the evolutionary differentiation and biogeographic pattern of *Leptochilus* and offers a more comprehensive scientific basis for the systematic revision and species diversity conservation of the genus. Future studies should expand the sampling scope of *Leptochilus* and integrate nuclear gene and plastid genome data to more comprehensively reveal the evolutionary history of *Leptochilus* species.

## Author Contributions

**Hua‐Jing Zhou:** data curation (lead), formal analysis (equal), investigation (equal), visualization (lead), writing – original draft (lead). **Ting Wang:** data curation (equal), formal analysis (lead), methodology (lead), visualization (equal), writing – original draft (supporting). **Yi Huang:** data curation (equal), investigation (equal). **Yu‐Han Fang:** data curation (equal), investigation (equal), supervision (equal). **Bin Zhang:** data curation (equal), formal analysis (equal). **Guo‐Di Chen:** investigation (lead). **Hong‐Feng Chen:** conceptualization (equal), methodology (equal), supervision (equal). **Fa‐Guo Wang:** conceptualization (lead), funding acquisition (lead), investigation (equal), supervision (lead), writing – review and editing (equal).

## Funding

This work was supported by the Guangdong Flagship Project of Basic and Applied Basic Research (2023B0303050001).

## Ethics Statement

The authors have nothing to report.

## Conflicts of Interest

The authors declare no conflicts of interest.

## Supporting information


**Data S1:** 44 samples of trnL‐F sequences.fasta.

**Data S2:** 45 samples of rbcL sequences.fasta.

**Data S3:** 46 samples of rps4‐trnS sequences.fasta.

## Data Availability

Type specimens of the new species described in this study are deposited at IBSC (1094808).
